# Supplementary material for: Time-series transcriptome analysis identified differentially expressed genes in broiler chicken infected with mixed Eimeria species
Source: Front Genet. 2022 Aug 8;13:886781. doi: 10.3389/fgene.2022.886781 (PMC9393255; doi:10.3389/fgene.2022.886781)
Supplement: Supplementary file 2 [file DataSheet1.ZIP › 7dpi_KEGG.Gsea.1625115961950/KEGG_CARDIAC_MUSCLE_CONTRACTION.html]

Details for gene set KEGG\_CARDIAC\_MUSCLE\_CONTRACTION[GSEA]

|  || Dataset | TMM\_7dpi\_gct\_format\_7dpi\_gct\_format.Class\_4dpi.cls #PC\_versus\_NC.Class\_4dpi.cls #PC\_versus\_NC\_repos |
| Phenotype | Class\_4dpi.cls#PC\_versus\_NC\_repos |
| Upregulated in class | 1 |
| GeneSet | KEGG\_CARDIAC\_MUSCLE\_CONTRACTION |
| Enrichment Score (ES) | 0.5298781 |
| Normalized Enrichment Score (NES) | 2.2066982 |
| Nominal p-value | 0.0 |
| FDR q-value | 0.001109465 |
| FWER p-Value | 0.0048 |
Table: GSEA Results Summary

  

Fig 1: Enrichment plot: KEGG\_CARDIAC\_MUSCLE\_CONTRACTION      
 Profile of the Running ES Score & Positions of GeneSet Members on the Rank Ordered List

  

| SYMBOL | TITLE | RANK IN GENE LIST | RANK METRIC SCORE | RUNNING ES | CORE ENRICHMENT || 1 | SLC9A1 | na | 79 | 1.313 | 0.0740 | Yes |
| 2 | COX5A | na | 300 | 0.942 | 0.1135 | Yes |
| 3 | UQCR10 | na | 444 | 0.785 | 0.1497 | Yes |
| 4 | COX6A1 | na | 480 | 0.760 | 0.1935 | Yes |
| 5 | UQCR11 | na | 485 | 0.754 | 0.2394 | Yes |
| 6 | UQCRB | na | 487 | 0.753 | 0.2856 | Yes |
| 7 | COX7A2 | na | 499 | 0.743 | 0.3303 | Yes |
| 8 | ACTC1 | na | 626 | 0.674 | 0.3611 | Yes |
| 9 | UQCRHL | na | 788 | 0.594 | 0.3842 | Yes |
| 10 | TPM4 | na | 862 | 0.566 | 0.4128 | Yes |
| 11 | COX6C | na | 930 | 0.546 | 0.4408 | Yes |
| 12 | COX7B | na | 1055 | 0.501 | 0.4612 | Yes |
| 13 | UQCRQ | na | 1103 | 0.484 | 0.4870 | Yes |
| 14 | COX4I1 | na | 1125 | 0.476 | 0.5145 | Yes |
| 15 | UQCRFS1 | na | 1263 | 0.437 | 0.5299 | Yes |
| 16 | UQCRC2 | na | 1700 | 0.332 | 0.5138 | No |
| 17 | COX7A2L | na | 1761 | 0.316 | 0.5282 | No |
| 18 | CACNB1 | na | 2104 | 0.252 | 0.5151 | No |
| 19 | ATP1B1 | na | 2312 | 0.220 | 0.5113 | No |
| 20 | UQCRC1 | na | 2326 | 0.217 | 0.5236 | No |
| 21 | RYR2 | na | 2775 | 0.154 | 0.4956 | No |
| 22 | CACNG1 | na | 2984 | 0.128 | 0.4861 | No |
| 23 | CACNB4 | na | 3215 | 0.099 | 0.4730 | No |
| 24 | TPM3 | na | 3456 | 0.068 | 0.4571 | No |
| 25 | SLC8A1 | na | 4125 | -0.009 | 0.4019 | No |
| 26 | CACNA2D1 | na | 4150 | -0.011 | 0.4006 | No |
| 27 | CACNA2D2 | na | 4152 | -0.012 | 0.4012 | No |
| 28 | TPM1 | na | 4614 | -0.062 | 0.3665 | No |
| 29 | COX7C | na | 4881 | -0.090 | 0.3498 | No |
| 30 | CACNB2 | na | 5132 | -0.116 | 0.3360 | No |
| 31 | CACNA1C | na | 5491 | -0.148 | 0.3152 | No |
| 32 | ATP1A1 | na | 5698 | -0.169 | 0.3084 | No |
| 33 | TPM2 | na | 5908 | -0.192 | 0.3027 | No |
| 34 | ATP2A2 | na | 6134 | -0.218 | 0.2973 | No |
| 35 | ATP1B4 | na | 7446 | -0.356 | 0.2096 | No |
| 36 | SLC9A6 | na | 9001 | -0.549 | 0.1136 | No |
| 37 | ATP1B3 | na | 9258 | -0.592 | 0.1285 | No |
| 38 | CACNA1D | na | 11943 | -1.649 | 0.0056 | No |
Table: GSEA details [plain text format]

  

Fig 2: KEGG\_CARDIAC\_MUSCLE\_CONTRACTION      
 Blue-Pink O' Gram in the Space of the Analyzed GeneSet

  

Fig 3: KEGG\_CARDIAC\_MUSCLE\_CONTRACTION: Random ES distribution      
 Gene set null distribution of ES for **KEGG\_CARDIAC\_MUSCLE\_CONTRACTION**
